# Food for thought: how nutrition impacts cognition and emotion

**DOI:** 10.1038/s41538-017-0008-y

**Published:** 2017-12-06

**Authors:** Sarah J. Spencer, Aniko Korosi, Sophie Layé, Barbara Shukitt-Hale, Ruth M. Barrientos

**Affiliations:** 1 School of Health and Biomedical Sciences, RMIT University, Melbourne, VIC 3788 Australia; 2 Swammerdam Institute for Life Sciences, Center for Neuroscience, University of Amsterdam, Amsterdam, 1098 XH Netherlands; 3 Nutrition et Neurobiologie Intégrée, INRA, Bordeaux University, Bordeaux, UMR1286 France; 4 USDA-ARS, Human Nutrition Research Center On Aging at Tufts University, Boston, MA 02111-1524 USA; 5 Department of Psychology & Neuroscience, and Center for Neuroscience, University of Colorado, Campus Box 345, Boulder, CO 80309-0345 USA

**Keywords:** Obesity, Neuroendocrine diseases

## Abstract

More than one-third of American adults are obese and statistics are similar worldwide. Caloric intake and diet composition have large and lasting effects on cognition and emotion, especially during critical periods in development, but the neural mechanisms for these effects are not well understood. A clear understanding of the cognitive–emotional processes underpinning desires to over-consume foods can assist more effective prevention and treatments of obesity. This review addresses recent work linking dietary fat intake and omega-3 polyunsaturated fatty acid dietary imbalance with inflammation in developing, adult, and aged brains. Thus, early-life diet and exposure to stress can lead to cognitive dysfunction throughout life and there is potential for early nutritional interventions (e.g., with essential micronutrients) for preventing these deficits. Likewise, acute consumption of a high-fat diet primes the hippocampus to produce a potentiated neuroinflammatory response to a mild immune challenge, causing memory deficits. Low dietary intake of omega-3 polyunsaturated fatty acids can also contribute to depression through its effects on endocannabinoid and inflammatory pathways in specific brain regions leading to synaptic phagocytosis by microglia in the hippocampus, contributing to memory loss. However, encouragingly, consumption of fruits and vegetables high in polyphenolics can prevent and even reverse age-related cognitive deficits by lowering oxidative stress and inflammation. Understanding relationships between diet, cognition, and emotion is necessary to uncover mechanisms involved in and strategies to prevent or attenuate comorbid neurological conditions in obese individuals.

## Introduction

**Fig. 1:**
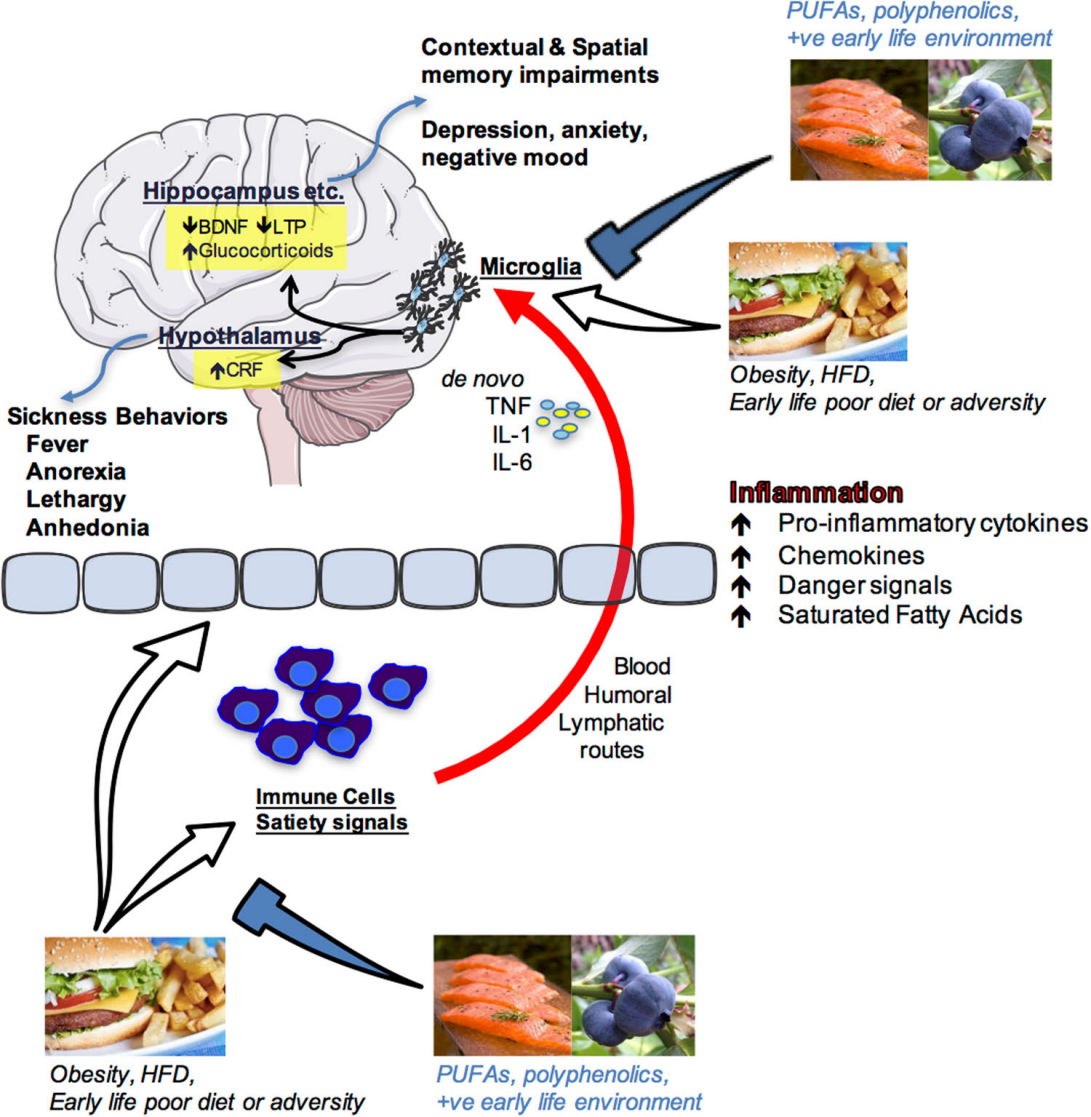
Schematic depiction of how nutrition influences cognition and emotion. Overeating, obesity, acute high-fat diet consumption, poor early-life diet or early life adversity can produce an inflammatory response in peripheral immune cells and centrally as well as having impact upon the blood–brain interface and circulating factors that regulate satiety. Peripheral pro-inflammatory molecules (cytokines, chemokines, danger signals, fatty acids) can signal the immune cells of the brain (most likely microglia) via blood-borne, humoral, and/or lymphatic routes. These signals can either sensitize or activate microglia leading to de novo production of pro-inflammatory molecules such as interleukin-1beta (IL1β), IL-6, and tumor necrosis factor alpha (TNFα) within brain structures that are known to mediate cognition (hippocampus) and emotion (hypothalamus, amygdala, prefrontal cortex and others). Amplified inflammation in these regions impairs proper functioning leading to memory impairments and/or depressive-like behaviors. Polyunsaturated fatty acids (PUFA), polyphenolics, and a positive (+ve) early life environment (appropriate nutrition and absence of significant stress or adversity) can prevent these negative outcomes by regulating peripheral and central immune cell activity. Images are adapted from Servier Medical Art, which is licensed under a Creative Commons Attribution 3.0 Unported License https://creativecommons.org/licenses/by/3.0/. Salmon and hamburger images were downloaded from Bing.com with the License filter set to “free to share, and use commercially”. The blueberry image is courtesy of author Assistant Prof. Ruth Barrientos

Cognitive and emotional dysfunctions are an increasing burden in our society. The exact factors and underlying mechanisms precipitating these disorders have not yet been elucidated. Next to our genetic makeup, the interplay between specific environmental challenges occurring during well-defined developmental periods seems to play an important role. Interestingly, such brain dysfunction most often co-occurs with metabolic disorders (e.g., obesity) and/or poor dietary habits; obesity and poor diet can lead to negative health implications including cognitive and mood dysfunctions, suggesting a strong interaction between these elements (Fig. 1). Obesity is a global phenomenon, with around 38% of adults and 18% of children and adolescents worldwide classified as either overweight or obese.^1^ Even in the absence of obesity, poor diet is commonplace,^2^ with, for instance, many eating foods that are highly processed and lacking in important polyphenols and anti-oxidants or that contain well-below the recommended levels of omega-3 polyunsaturated fatty acids (PUFA). In this review, we will discuss the extent of, and mechanisms for, diet’s influence on mood and cognition during different stages of life, with a focus on microglial activation, glucocorticoids and endocannabinoids (eCBs).

## Perinatal diet disrupts cognitive function long-term, a role for microglia

Poor diet in utero and during early postnatal life can cause lasting changes in many aspects of metabolic and central functions, including impairments in cognition and accelerated brain aging,^3^ but see.^4^ Maternal gestational diabetes and even a junk food diet in the non-diabetic can lead to metabolic complications, including diabetes and obesity in the offspring.^5,6^ It can also cause changes in reward processing in the offspring brain such that they grow to prefer foods high in fat and sucrose.^7,8^ Similarly, early introduction of solid food in children and high childhood consumption of fatty foods and sweetened drinks can accelerate weight gain and lead to metabolic complications long-term that may be associated with poorer executive function.^9^ On the other hand, some dietary supplements can positively influence cognition, as is seen with supplementation of baby formula with long chain omega-3 PUFA improving cognition in babies.^10^ In these randomized control trials (RCTs), an omega-3 PUFA-enriched formula beginning shortly after birth, or 6 weeks’ breast feeding, significantly improved performance of 9-month old babies on a problem solving task (a two-step task to retrieve a rattle, known to correlate with performance on IQ tasks).

From animal models, it is clear that the effects of diet in early life are far-reaching. Even obesity in rat sires (that play no part in rearing the offspring) leads to pancreatic beta cell dysfunction in female offspring, which can be passed on to the next generation.^11^ Obesity and high-fat diet feeding in rat and mouse dams during pregnancy and lactation leads to impairments in several tests of mood, including those modeling depressive and anxious behaviors, as well as negatively impacting cognition.^12^ Diet in the post-partum to weaning period can impact similar behaviors.^13^

Additional to the impact of a prenatal diet, over-consumption of the mother’s milk during the first 3 weeks of a rat’s life leads to lasting obesity in males and females.^14^ This neonatal overfeeding also disrupts cognitive function. For example, neonatally overfed rats perform poorly in the novel object recognition test and in the delayed spatial win-shift radial arm maze, as adults, compared with control rats.^15^ These findings are interesting to compare with the effects of poor diet in adults where a longer-term high-fat diet (around 20 weeks in the rat)^16–18^ and / or high-fat diet in conjunction with a pre-diabetic phenotype^19^ is necessary to induce cognitive dysfunction. While there are no differences in post-learning synaptogenesis (synaptophysin) or apoptosis (caspase-3) to explain the effects seen in the neonatally overfed, these rats do have an impaired microglial response to the learning task.^15^

Microglia are one of the major immune cell populations in the brain. In development, they are essential for synaptic pruning, while in a mature animal their major role is in mounting a pro-inflammatory immune response and phagocytosing pathogens and injured brain cells.^20^ Hyper-activated microglia can lead to cognitive dysfunction through excess pro-inflammatory cytokine production causing impaired long-term potentiation-induction, reduced production of plasticity-related molecules including brain-derived neurotrophic factor and insulin-like growth factor-1, and reduced synaptic plasticity^20^ However, an appropriate microglial response may also be essential for effective learning.

Neonatally overfed rats have more microglia in the CA1 region of the hippocampus at postnatal day 14, i.e., while they still have access to excess maternal milk and are undergoing accelerated weight gain. These microglia also have larger soma and retracted processes, indicative of a more activated phenotype. By the time these rats reach adulthood, there persists an increase in the area immunolabelled with microglial marker Iba1 in the dentate gyrus. In the neonatally overfed, the microglial response to a learning task is less robust than in controls. This effect is associated with a suppression of cell proliferation in control animals relative to the neonatally overfed, potentially to preserve existing neuronal networks and minimize novel inputs while learning takes place.^21^ Interestingly, global inducible microglial and monocyte depletion can lead to improved performance in the Barnes maze,^22^ suggesting withdrawal of microglial activity at specific learning phases is important for learning. These findings implicate microglia in the long-term effects of early life overfeeding on cognition suggesting normal microglia must be able to robustly respond to learning tasks and neonatal overfeeding impairs their ability to do so.

Neuroinflammatory processes, including the role of microglia, can clearly be impacted by neonatal diet and represent at least one contributing mechanism for how cognitive function is affected. Neuroinflammation and microglia can also be impacted by other early life events and play a significant role in how stress during development alters long-term physiology.

## Early-life stress (ES) programs vulnerability to cognitive disorders

ES alters brain structure and function life-long, leading to increased vulnerability to develop emotional and cognitive disorders as is evident from several preclinical and clinical studies.^23–25^ The exact underlying mechanisms for such programming remain elusive. There is extensive seminal work indicating a key role for sensory stimuli from the mother and neuroendocrine factors (e.g., stress hormones) in this programming,^26,27^ however it has been recently suggested that these factors might act synergistically with metabolic and nutritional elements.^28^ In fact, ES is associated with increased vulnerability to develop metabolic disorders such as obesity, which mostly co-occur with cognitive deficits,^29,30^ and both ES and an adverse early nutritional environment lead to strikingly similar cognitive impairments later in life,^28,31^ suggesting that metabolic factors and nutritional elements might mediate some of the ES effects on brain structure and function.

The brain has a very high demand for nutrients in this early period and nutritional imbalances affect normal neurodevelopment resulting in lasting cognitive deficits.^32^ Understanding the role of metabolic factors and specific nutrients in this context is key to develop effective peripheral (e.g., nutritional) intervention strategies. A mouse model of the chronic ES of limited nesting and bedding material during the first postnatal week has been shown to lead to aberrant maternal care, which leads to cognitive decline in the ES offspring.^24,33,34^

The hippocampus, a brain region key for cognitive functions, is permanently altered in its structure and function in these ES-exposed offspring. The hippocampus is in fact particularly sensitive to the early-life environment as it continues its development into the postnatal period.^35^ Adult neurogenesis (AN) is a unique form of plasticity, which takes place in the hippocampus, consisting of the proliferation of neuronal progenitor cells that differentiate and mature into fully functional neurons that subsequently integrate into the existing hippocampal circuitry. These newly formed neurons are involved in various aspects of hippocampus-dependent learning and memory.^36^ AN is affected persistently by ES^24,37^ and, more precisely, while ES exposure initially increases neurogenesis (i.e., proliferation and differentiation of newborn cells) at postnatal day 9, at later time points (postnatal day 150), the survival of the newly born cells is reduced.^24^ In addition, ES affects the neuroinflammatory profile in a lasting manner, with, for example, increased CD68 (phagocytic microglia expression) in adulthood.^38^

Importantly, ES persistently affects peripheral adipose tissue metabolism as well. White adipose mass (WAT), plasma leptin (the adipokine released from the WAT) and leptin mRNA expression in WAT are persistently reduced in ES-exposed offspring.^39^ In addition, exposure of ES mice to an unhealthy western style diet, leads to a higher increase in adiposity in these mice when compared to controls. These findings suggest that ES exposure leads to metabolic dysregulation and a greater vulnerability to develop obesity in a moderately obesogenic environment. Whether these metabolic alterations contribute to the ES-induced cognitive deficits warrants further investigation.^39^

In addition to peripheral metabolism, ES-induced alterations in the nutritional composition of the dam’s milk, and/or nutrient intake/absorption by the pup^25,28,40^ could have lasting consequences for brain structure and function. Indeed, the essential micronutrient, methionine, a critical component of the one-carbon (1-C) metabolism that is required for methylation, and for synthesis of proteins, phospholipids and neurotransmitters, is reduced after ES exposure in plasma and hippocampus of postnatal day 9 offspring. Importantly, a short supplementation of the maternal diet only during ES exposure with essential 1-C metabolism-associated micronutrients not only restores methionine levels peripherally as well as centrally, but rescues (some of) the effects of ES on hippocampal cognitive measures in adulthood and prevents the ES-induced hypothalamic-pituitary–adrenal axis hyperactivity at postnatal day 9.^25^

These studies highlight the importance of studying metabolic factors and nutrients in the ES-induced effects on the brain. In the near future, it will be key to further understand the exact mechanisms mediating the effects of nutrients and metabolic factors and the windows of opportunity for interventions on brain function, as this will open entirely new avenues for targeted nutrition for vulnerable populations. However, while the early life period is a window of particular vulnerability to the programming effects of diet and other environmental influences, diet at other phases of life is also important in dictating mood and cognition.

## Adult consumption of a high-fat diet: a vulnerability factor for hippocampal-dependent memory

Adults in developed countries are consuming diets higher in saturated fats and/or refined sugars than ever before. Indeed, recent reports show that approximately 12% of American adults’ daily energy intake comes from saturated fats and 13% from added sugars,^41^ significantly more than what is recommended (5–10%) by the US Department of Agriculture and the Department of Health and Human Services. Not surprisingly, these dietary habits have contributed to the increasing prevalence of obesity among adults, which is currently approximately 37% in the US, a sharp rise from the 13% prevalence rate of 1960.^42^

These statistics are alarming because aside from its well-known provocation of cardiovascular disease, metabolic syndrome, and type 2 diabetes, obesity has now also been associated with mild cognitive impairments and dementia. There is growing evidence that neuroinflammation may underlie obesity-induced cognitive deficits.^9^ Recently, studies have demonstrated that short-term consumption (1–7 days) of an unhealthy diet (e.g., high saturated fat and/or high sugar) triggers neuroinflammatory processes, suggesting that obesity per se may not be necessary to cause cognitive disruptions.^43,44^ For the last 10–15 years, the hypothalamus has received the vast majority of the attention with regard to obesity-induced neuroinflammatory responses and functional declines,^45^ perhaps due to its close proximity to the third ventricle, circumventricular organs, and mediobasal eminence, where inflammatory signals from the periphery have easier entry into the brain. Indeed, long chain saturated fatty acids have been shown to directly pass into the hypothalamus producing an inflammatory response there through activation of toll-like receptor 4 signaling.^46,47^ This active passage of saturated fatty acids, however, has not been observed in the hippocampus, a key brain region that mediates learning and memory.^46^ Nonetheless, high-fat diet consumption has been demonstrated to impair hippocampus-dependent memory function in humans and rodents. For example, compared to rodents that consumed a control diet, those that consumed a high-fat and/or high-sugar diet exhibited robust impairments in various types of memory (e.g., spatial, contextual), as indicated by weaker performances in the Y-maze,^48^ radial arm maze,^15^ novel object recognition task,^15^ novel place recognition task,^44,49^ Morris water maze,^50^ and contextual fear conditioning.^18,51^ Also, adult humans who consumed a high-fat diet for 5 days exhibited significantly reduced focused attention and reduced retrieval speed of information from working and episodic memory, compared with those who consumed a standard diet.^52^

Many of these studies, and others, have shown that high-fat diet-induced cognitive deteriorations are accompanied by elevated neuroinflammatory markers or responses in the hippocampus.^15,18,44,48–51,53^ However, the mechanisms by which these neuroinflammatory processes signal and/or affect the hippocampus are not entirely clear. There is growing evidence that high-fat diets may compromise the hippocampus by sensitizing the immune cells (most likely microglia) of this brain structure, thus priming the inflammatory response to subsequent challenging stimuli.^18,50,51^ For example, one study demonstrated that adult rats that had eaten a high-fat diet for 5 months exhibited a sensitized hippocampus such that when they received a relatively mild stressor (a single, 2 s, 1.5 mA footshock) following a learning session the neuroinflammatory response in the hippocampus was potentiated compared to the response of rats that had eaten the regular chow, and this response led to deficits in long-term contextual memory.^18^ Another study showed that just 3 days of consuming a high-fat diet was sufficient to sensitize the hippocampus of adult rats. Here, a low-dose peripheral immune challenge (with lipopolysaccharide; LPS) produced an exaggerated neuroinflammatory response in the hippocampus of these rats compared to those that consumed the regular chow, and also led to contextual memory deficits.^51^

Significantly elevated pro-inflammatory cytokines in the hippocampus have been shown to deteriorate various mechanisms that enable synaptic plasticity (such as long-term potentiation), and thus long-term memory.^54^ Sobesky et al.^51^ demonstrated that high-fat diet consumption primes the cells of the hippocampus by elevating the glucocorticoid steroid hormone corticosterone in this region. Despite its classic role as an immunosuppressant, there is increasing evidence demonstrating that corticosterone can prime hippocampal microglia and potentiate the inflammatory response to a subsequent challenge.^55–57^ For example, Frank et al.^55^ elegantly showed that when corticosterone was elevated prior to a peripheral immune challenge (LPS), the resulting inflammatory response in the hippocampus was potentiated. In contrast, when corticosterone was elevated after the immune challenge, the neuroinflammatory response was suppressed. These findings suggest that the temporal relationship between the corticosterone increase and the immune challenge dictates whether a pro-inflammatory or anti-inflammatory response will result.^55^ Sobesky et al.^51^ found that rats that consumed the high-fat diet for 3 days exhibited significantly increased levels of corticosterone in their hippocampus compared to rats that consumed the regular chow or a novel macronutrient-matched control diet. This high-fat diet-induced corticosterone rise was accompanied by increases in the endogenous danger-associated molecular pattern high-mobility group box 1 (HMGB1), the interleukin (IL)-1 inflammasome-associated protein NLRP3, and the microglial activation marker cd11b. high-fat diet alone did not, however, elevate the pro-inflammatory cytokine IL-1β unless rats were subsequently challenged with a low-dose of LPS. Thus, LPS challenge potentiated the pro-inflammatory response in the hippocampus of high-fat diet-fed rats compared to the response to LPS in chow-fed rats. To evaluate the role of corticosterone signaling in neuroinflammatory priming caused by consumption of high-fat diet, Sobesky et al.^51^ administered the glucocorticoid receptor antagonist, mifepristone, prior to high-fat diet consumption. This resulted in a normalized hippocampal IL-1β response to low-dose LPS. Furthermore, mifepristone significantly reduced the high-fat diet + LPS-induced expression of HMGB1, IκBα, and NLRP3. Moreover, mifepristone treatment effectively prevented contextual memory deficits caused by high-fat diet consumption combined with LPS challenge. These data provide strong evidence for the idea that (a) high-fat diet consumption increases corticosterone within the hippocampus, and (b) this corticosterone is a key mediator in sensitizing microglia or other immune cells of the hippocampus; (c) sensitized microglia produce a potentiated neuroinflammatory response to subsequent immune or stressful challenges, thus producing cognitive deficits. Notably, though, while high-fat diet per se can have significant detrimental impact on cognitive processes, specific dietary components may be able to reverse these effects, omega-3 PUFA are one such potentially beneficial component.

## Dietary omega-3 PUFA regulate neuroinflammation and eCBs: role in mood and cognitive disorders

Since their discovery in the early 20th century, considerable attention has been paid to the roles of PUFA in brain functions. Omega-3 and omega-6 PUFA are essential fatty acids, meaning that they have to be provided by the diet. Western diet contains excessive amounts of omega-6 PUFA as compared to omega-3 leading to an unbalanced ratio between these two fatty acids with cardiovascular and brain health consequences. Essential omega-3 and omega-6 fatty acids are found in green vegetables, seeds and nuts although coming from different sources with linolenic acid (LA, 18:2 omega-6) found in most plants, coconut and palm and α-linolenic acid (ALA, 18:3 omega-3) in green leafy vegetables, flax and walnuts. Once consumed, LA and ALA are metabolized into arachidonic acid (AA, 20:4 omega-6) and docosahexaenoic acid (DHA, 22:6 omega-3), respectively.

AA and DHA are the main omega-6 and omega-3 long chain PUFA found in the brain. Both long chain PUFA have pivotal roles in brain physiology as they regulate fundamental neurobiological processes, in particular the ones involved in cognition and mood.^58,59^ AA and DHA are esterified to the phospholipid of neuronal and glial cell membranes with a total brain phospholipid proportion of around 10% for AA and 20% for DHA. Due to the limited capacity of the brain to synthesize long chain PUFA, preformed DHA can be provided by dietary supply of oily fishes. Hence, increased consumption of DHA-rich products results in a partial replacement of AA by DHA in brain cell membranes.^60^ Conversely, a lower omega-3 PUFA intake leads to lower brain levels of DHA with increased AA levels. Higher AA and DHA are reported in women as compared to men, suggesting a gender difference in PUFA levels.^61^ These differences could be linked to sex hormones as they differentially influence PUFA metabolism with estrogen stimulating, and testosterone inhibiting, the conversion of both omega-3 and omega-6 precursors into their respective long chain metabolites. However, whether these differences in PUFA have a role in specific brain diseases with a gender component has been poorly questioned and requires further investigation.

After its direct consumption and/or metabolization in the liver, DHA is increased in the blood and is likely to freely enter into the brain as non-esterified fatty acid.^58^ More recently, Mfsd2a (major facilitator superfamily domain-containing protein 2a), which is expressed by brain endothelial cells and adiponectin receptor 1 in the retina, has been revealed to be important to DHA uptake and retention.^62^

Abnormal omega-3 PUFA levels have been extensively described in both the peripheral tissues and in the brain of patients with mood disorders or cognitive decline, leading to a large number of RCTs aiming at evaluating the effectiveness of long chain omega-3 PUFA dietary supplementation on mood and cognitive disorders.^58,63^ Overall, the results are discordant, due to the heterogeneity of methods used to evaluate the depressive and/or cognitive symptoms, the form, dose and duration of the omega-3 PUFA supplementation, the lack of evaluation of nutritional intake and metabolism of PUFA prior to starting the supplementation, or the lack of evaluation of genotype-associated risk factors.^64^ However, despite the discrepancies in the results, it is important to note that several RCTs performed in patients with depressive disorders revealed an additional effect of long chain omega-3 PUFA supplementation to antidepressant treatments.^65^ Of note, a recent study identifies that depressive patients presenting a high level of inflammatory markers are more responsive to long chain omega-3 PUFA supplementation.^66^ This observation is highly relevant as these PUFA are potent regulators of inflammation^58^ and inflammation is a crucial component of mood disorders. Concerning cognitive decline, despite poor positive results of PUFA dietary supplementation in Alzheimer’s disease (AD) patients, RCTs using DHA supplementation in subjects carrying the apolipoprotein E ε4 (APOE4) allele, a risk factor for AD, reveal an improvement of pre-dementia.^64^ Overall, discrepancies in clinical studies strongly support the need for preclinical studies aimed at depicting the mechanisms of omega-3 PUFA on brain dysfunctions, which should help to better target populations at risk of cognitive and mood disorders. In addition, the consideration of omega-3 PUFA levels in food to cover the physiological requirement of these PUFA for an optimal brain function is a challenge for the food industry.

Through direct or indirect effects, DHA and AA modulate neurotransmission and neuroinflammation, which are key processes in cognition and mood.^58,59^ Unesterified long chain PUFA are released from cell membranes upon the activation of phospholipase A2 (PLA2) to exert their effects.^67^ Once released, AA and DHA are metabolized into bioactive mediators through cyclooxygenase (COX), lipoxygenases (LOX) and cytochrome P450.^68^ The conversion of AA into several prostanoids, including prostaglandins (PG), leukotrienes (LT), thromboxanes (TX) and lipoxins (LX), is crucial in the progression of inflammation, including in the brain.^58^ DHA is also metabolized through the COX/LOX pathways to generate metabolites with anti-inflammatory and pro-resolutive properties.^68^ In the brain, LOX-derived specialized proresolving mediators (SPMs), neuroprotectin D1 (NPD1), resolvin D5 (RvD5), and maresin 1 (MaR1) are detected.^68,69^ Some of these SPMs potently modulate neuroinflammation in vivo and in vitro, through their direct effect on microglia.^70,71^ DHA and SPMs are impaired at the periphery and in the brains of AD patients.^72,73^ Interestingly, decreased DHA distribution in AD patient brains correlates with synaptic loss rather than amyloid beta (Aβ) deposition.^74^ In addition, DHA or SPMs promote phagocytosis of Aβ42 by microglia^75^ and modulate microglia number and activation in vivo.^76^ Whether SPMs play a role in the protective activity of long chain omega-3 PUFA in mood and cognitive disorders associated to neuroinflammation remains to be established.

eCBs are other key PUFA-derived lipid mediators in the brain. The main brain AA-derived eCBs are the fatty acid ethanolamides anandamide (AEA) and 2-arachidonoylglycerol (2-AG), while docosahexaenoylethanolamide (DHEA or synaptamide) is an eCB-like derived from DHA.^77^ ECBs half-life in the brain is regulated by specific catabolizing enzymes fatty acid amide hydrolase for AEA and DHEA and monoacylglycerol lipase for 2-AG. Regarding neuroinflammatory processes, AA-derived eCBs are oxidized into bioactive PG by COX and LOX, which promote inflammation.^78^ AEA and 2-AG bind to at least two cannabinoid receptors, type 1 (CB1) and type 2 (CB2), which are Gi/o protein-coupled with numerous signaling pathways in the brain.^79,80^ DHEA has a lower binding affinity for CB1 and CB2 receptors as compared to AEA and 2-AG and rather bind GPR receptors, in particular GPR110 in the brain. The dietary omega-3/omega-6 PUFA ratio directly influences the proportion of ethanolamides derived from AA and DHA.^81^ The modulation of eCB is accompanied by the impairment of neuronal CB1R activity and synaptic activity in several brain structures.^82,83^ 2-AG and AEA regulate synaptic function by suppressing excitatory and inhibitory synapse neurotransmitter release by acting as retrograde messengers at presynaptic CB1.^84^ The importance of brain eCB signaling in the understanding of how altered dietary intake of PUFA correlates with a range of neurological disorders is of high interest.^81^ However, other dietary factors may also contribute to improved cognition and prevention of cognitive disorders. Polyphenolic-rich foods are a further example that have been shown to have benefit, particularly in the context of aging.

## Dietary interventions with polyphenolic-rich foods can improve neuronal and behavior deficits associated with aging

It is estimated that approximately 20% of the US total population will be older than 65 by the year 2050, which is almost double what it is today.^85^ Additionally, the US is faced with an increasingly overweight/obese population that is at heightened risk for metabolic disorders, resulting in diabetes and cardiovascular disease, and concomitant behavioral impairment. Aging and metabolic dysregulation are both associated with numerous cognitive and motor deficits on tasks that require fine motor control, balance, short-term and long-term memory, or executive function. Studies in both humans and animal models have demonstrated that oxidative stress and inflammation, as well as impaired insulin resistance, are common features in cardio-metabolic and vascular disease, obesity, and age-related declines in cognitive and motor function.^86^ Neuroinflammation occurs locally in the brain; however, peripheral inflammatory cells and circulating inflammatory mediators (e.g., cytokines) can also infiltrate the brain, and this occurs more readily as we age.^87^ Therefore, strategies must be found to reduce oxidative and inflammatory vulnerability to age-related changes and reverse deficits in motor and cognitive function.

Targeting peripheral inflammation and insulin signaling could reduce insulin resistance and infiltration of inflammatory mediators into the brain and, as a result, reduce the incidence of a variety of age-related deficits. Studies have shown that plants, particularly colorful fruit or vegetable-bearing plants, contain polyphenolic compounds that have potent antioxidant and anti-inflammatory activities,^88^ and increased fruit and vegetable intake has been associated with reduced fasting insulin levels.^89^ Evidence is accumulating that consumption of these polyphenol-rich foods, particularly berry fruit, may be a strategy to forestall or even reverse age-related neuronal deficits resulting from neuroinflammation.^90^ Recently this evidence has been extended to double-blind, placebo-controlled, randomized human intervention studies that have demonstrated that the consumption of flavonoid/polyphenols is associated with benefits to cognitive function.^91^

Preclinical studies have led to the hypothesis that the key to reducing the incidence of age-related deficits in behavior is to alter the neuronal environment with polyphenolic-rich foods like berry fruit, such that neuroinflammation and oxidative stress, and the vulnerability to them, would be reduced. In early studies with animal models, crude blueberry (BB) or strawberry extracts significantly attenuated^92^ and reversed^93^ age-related motor and cognitive deficits in senescent rodents. BB supplementation also protected 9 month old C57Bl/6 mice against the damaging effects of consuming a high-fat diet.^94^ Novel object recognition memory was impaired by the high-fat diet, but blueberry supplementation prevented recognition memory deficits in a time-dependent manner. Spatial memory, as measured by the Morris water maze, was also improved after 5 months on the diets.^94^ Subsequent research suggested that berry fruit polyphenols may possess a multiplicity of actions in addition to their anti-inflammatory and antioxidant activities.^90^ Additionally, the anthocyanins contained in blueberries have been shown to enter the brain, and their concentrations were correlated with cognitive performance.^95^

Epidemiological studies that have focused on fruit and vegetable intake and cognitive function have also largely found that adequate consumption can prevent cognitive decline, while low intake is associated with increased cognitive decline.^85^ Specifically, increased intake of blueberries and strawberries, as well as increased intakes of anthocyanidins and total flavanoids, were associated with slowing the rate of cognitive decline by up to 2.5 years.^96^

The ability of berry fruit to protect against age-related cognitive decline has also been examined in a growing number of double-blind, placebo-controlled, randomized, human intervention studies. Thus, blueberry juice significantly improved word list recall and paired associate learning in older men and women with age-related memory decline that consumed it, relative to baseline, with paired associate learning also significantly improved relative to placebo controls.^97^ A recent study^98^ that measured similar cognitive tasks as those in the rodent studies, showed that freeze-dried blueberries (24 g/day, equivalent to one cup of fresh blueberries) for 90 days improved two measures of executive function in older adults (ages 60–75). Participants in the blueberry group showed significantly fewer repetition errors in the California Verbal Learning test as well as reduced switch cost on a task-switching test across study visits, relative to controls who consumed placebo powder. However, no improvement in gait or balance was observed following blueberry intake.^98^ Finally, 12 weeks of blueberry concentrate supplementation improved brain perfusion, task-related activation, and cognitive function (i.e., working memory) in healthy older adults who consumed 30 mL blueberry concentrate providing 387 mg anthocyanidins.^99^ These studies suggest that berry fruit might be an effective strategy to prevent, delay, or reverse cognitive dysfunction during aging.

Cognitive aging does not occur simultaneously across cognitive domains, with various domains peaking in early adulthood before reaching a plateau or declining. Therefore, interventions early in life may yield health benefits that are only measureable in later life. Blueberries have been shown to have positive cognitive benefits in two acute, cross-over designed studies in school-aged children (ages 7–10). The first study^100^ showed that consumption of a flavonoid-rich blueberry (200 g) drink led to significantly better delayed word-list recall, compared to a matched vehicle group, on the Rey auditory-verbal learning test, suggesting more effective coding of memory items. However, there was no benefit of blueberry intervention on measures of attention, response inhibition, or visuospatial memory, and a negative impact on proactive interference.^100^ The second study^100^ by the same group examined cognition at baseline, and then 1.15, 3, and 6 h after consuming placebo (vehicle) or blueberry drinks containing 15 or 30 g freeze-dried wild blueberry (WBB) powder. Consumption of WBB powder improved recall at 1.15 h, improved delayed word recognition, which was sustained at each time point measured, and improved accuracy on a challenging interference task at 3 h. The best cognitive performance was seen after the 30 g dose, and particularly on those tasks with a higher cognitive demand.^100^

As humans age, their ability to defend against the effects of oxidative stress and inflammation weakens, putting elderly people at increased risk for neuronal disease and degradation. Neuroprotective foods, such as berries and other dark-colored fruits, represent one way to protect aging brains against this damage by reducing inflammation and oxidative stress in the brain, thereby protecting against cognitive declines in aged populations.

## Conclusion

This review has highlighted the latest advances in how foods and patterns of consumption at different times of development affect the brain, and the behavioral manifestations that may result from these effects. For example, early life overfeeding can permanently sensitize the brain’s neuroinflammatory response to challenging stimuli resulting in cognitive and immune dysfunctions throughout life. ES alters brain function, via metabolic and nutritional factors, to increase vulnerability to develop emotional and cognitive disorders. Long-term and short-term consumption of high saturated fatty foods during adulthood produces a sensitized inflammatory phenotype, via a glucocorticoid rise, in the hippocampus, leading to learning and memory vulnerabilities. Imbalance of omega-3 and omega-6 PUFA contribute to neurodevelopmental disorders by altering microglial activation resulting in abnormal formation of neuronal networks and activity. Finally, consumption of fruits and vegetables high in polyphenolics can prevent and reverse age-related cognitive deficits by lowering oxidative stress and inflammation. Collectively these data show that attention to dietary composition is important for lasting impact beyond the metabolic and highlight the promising likelihood that we may improve our cognition throughout life and into the aging period with simple dietary interventions. These data highlight the need for food industries and science, alike, to focus on research and development of nutritional strategies that are most appropriate to support our cognitive and emotional health; foods that are high in omega-3 PUFA and polyphenolics may be a promising place to start.

### Data availability

No data sets were generated or analyzed during the current study.
